# The prognostic value of lymph node yield in the earliest stage of colorectal cancer: a multicenter cohort study

**DOI:** 10.1186/s12916-017-0892-7

**Published:** 2017-07-14

**Authors:** Yara Backes, Sjoerd G. Elias, Bibie S. Bhoelan, John N. Groen, Jeroen van Bergeijk, Tom C. J. Seerden, Hendrikus J. M. Pullens, Bernhard W. M. Spanier, Joost M. J. Geesing, Koen Kessels, Marjon Kerkhof, Peter D. Siersema, Wouter H. de Vos tot Nederveen Cappel, Niels van Lelyveld, Frank H. J. Wolfhagen, Frank ter Borg, G. Johan A. Offerhaus, Miangela M. Lacle, Leon M. G. Moons, on behalf of the Dutch T1 CRC Working Group

**Affiliations:** 1 Department of Gastroenterology & Hepatology, University Medical Center Utrecht, Heidelberglaan 100, 3508 GA Utrecht, The Netherlands; 2 Julius Center for Health Sciences and Primary Care, University Medical Center Utrecht, Utrecht, The Netherlands; 3 Department of Gastroenterology & Hepatology, Sint Jansdal Hospital, Harderwijk, The Netherlands; 4 Department of Gastroenterology & Hepatology, Gelderse Vallei Hospital, Ede, The Netherlands; 5 Department of Gastroenterology & Hepatology, Amphia Hospital, Breda, The Netherlands; 6 Department of Gastroenterology & Hepatology, Meander Medical Center, Amersfoort, The Netherlands; 7 Department of Gastroenterology & Hepatology, Rijnstate Hospital, Arnhem, The Netherlands; 8 Department of Gastroenterology & Hepatology, Diakonessenhuis, Utrecht, The Netherlands; 9 Department of Gastroenterology & Hepatology, Flevo Hospital, Almere, The Netherlands; 10 Department of Gastroenterology & Hepatology, Groene Hart Hospital, Gouda, The Netherlands; 11 Department of Gastroenterology & Hepatology, Radboud University Medical Center, Nijmegen, The Netherlands; 12 Department of Gastroenterology & Hepatology, Isala Hospital, Zwolle, The Netherlands; 13 Department of Gastroenterology & Hepatology, Sint Antonius Hospital, Nieuwegein, The Netherlands; 14 Department of Gastroenterology & Hepatology, Albert Schweitzer Hospital, Dordrecht, The Netherlands; 15 Department of Gastroenterology & Hepatology, Deventer Hospital, Deventer, The Netherlands; 16 Department of Pathology, University Medical Center Utrecht, Utrecht, The Netherlands

**Keywords:** Malignant polyps, T1 colorectal carcinoma, Lymph node metastasis, Lymph node retrieval

## Abstract

**Background:**

In patients with stage II colorectal cancer (CRC) the number of surgically retrieved lymph nodes (LNs) is associated with prognosis, resulting in a minimum of 10–12 retrieved LNs being recommended for this stage. Current guidelines do not provide a recommendation regarding LN yield in T1 CRC. Studies evaluating LN yield in T1 CRC suggest that such high LN yields are not feasible in this early stage, and a lower LN yield might be appropriate. We aimed to validate the cut-off of 10 retrieved LNs on risk for recurrent cancer and detection of LN metastasis (LNM) in T1 CRC, and explored whether this number is feasible in clinical practice.

**Methods:**

Patients diagnosed with T1 CRC and treated with surgical resection between 2000 and 2014 in thirteen participating hospitals were selected from the Netherlands Cancer Registry. Medical records were reviewed to collect additional information. The association between LN yield and recurrence and LNM respectively were analyzed using 10 LNs as cut-off. Propensity score analysis using inverse probability weighting (IPW) was performed to adjust for clinical and histological confounding factors (i.e., age, sex, tumor location, size and morphology, presence of LNM, lymphovascular invasion, depth of submucosal invasion, and grade of differentiation).

**Results:**

In total, 1017 patients with a median follow-up time of 49.0 months (IQR 19.6–81.5) were included. Four-hundred five patients (39.8%) had a LN yield ≥ 10. Forty-one patients (4.0%) developed recurrence. LN yield ≥ 10 was independently associated with a decreased risk for recurrence (IPW-adjusted HR 0.20; 95% CI 0.06–0.67; *P* = 0.009). LNM were detected in 84 patients (8.3%). LN yield ≥ 10 was independently associated with increased detection of LNM (IPW-adjusted OR 2.27; 95% CI 1.39–3.69; *P* = 0.001).

**Conclusions:**

In this retrospective observational study, retrieving < 10 LNs was associated with an increased risk of CRC recurrence, advocating the importance to perform an appropriate oncologic resection of the draining LNs and diligent LN search when patients with T1 CRC at high-risk for LNM are referred for surgical resection. Given that both gastroenterologists, surgeons and pathologists will encounter T1 CRCs with increasing frequency due to the introduction of national screening programs, awareness on the consequences of an inadequate LN retrieval is of utmost importance.

**Electronic supplementary material:**

The online version of this article (doi:10.1186/s12916-017-0892-7) contains supplementary material, which is available to authorized users.

## Background

With the introduction of colorectal cancer (CRC) screening programs, there has been a shift towards an increased detection of T1 CRC as compared to more advanced cancers [1, 2]. Endoscopic resection is considered curative for low-risk T1 CRC. However, surgical resection is recommended for patients with T1 CRC with a considerable risk of lymph node metastasis (LNM), as determined by histological high-risk factors [3, 4]. The prognosis of these patients depends to a large extent on the lymph node (LN) status, with a CRC-related 5-year survival of ≥ 95% in the absence of LNM (American Joint Committee on Cancer (AJCC) stage I T1 CRC), decreasing to 68–90% in the presence of LNM (AJCC stage III T1 CRC) [5, 6].

In contrast to AJCC stage II CRC (T3–4 CRC), in which a higher LN yield has been associated with improved survival, little is known on the association between LN yield and recurrence in T1 CRC [7, 8]. Therefore, international guidelines restrict their recommendation for a minimum yield of 10–12 LNs to AJCC stage II CRC [9–12]. It is questionable whether the recommended cut-off in advanced CRC can be extrapolated to T1 CRC, given that more advanced tumor depth has been associated with an increased LN yield [13–15]. Accordingly, reported LN yields in T1 CRC are much lower, with mean and median LN yields between 4 and 7 in studies performed between 1988 and 2003 [13, 16–18]. One might hypothesize that the additional value of retrieving more LNs might be less relevant for T1 CRCs as LNM are reported in only 8–12% of patients and recurrence after surgical resection is reported in only 2–5% of patients [3, 4, 19]. Two small single-center studies argued that a minimum of 4 and 8 retrieved LNs, respectively, should be appropriate for staging T1 CRC [16, 20], whereas La Torre et al. [21] concluded that a limited resection does not affect oncological outcome in this early stage. However, these studies were either small or not informed about long-term recurrence rates.

In this longitudinal multicenter retrospective cohort study consisting of patients who underwent surgical resection of T1 CRC, we aimed to explore the association between LN yield and the risk for recurrence, and to assess the association between LN yield and the detection of LNM. Moreover, we aimed to explore whether a minimum of 10 LNs is feasible in a routine clinical setting.

## Methods

### Patients and study design

This is a multicenter retrospective cohort study. Patients diagnosed with T1 CRC in 13 participating hospitals (1 academic and 12 non-academic hospitals) between 1 January 2000 and 31 December 2014 were selected from the Netherlands Cancer Registry. The electronic medical records of all patients were reviewed. Only cases in which the local pathologist clearly confirmed the diagnosis T1 CRC in the pathology report were selected, which was defined as tumors with invasion through the muscularis mucosa and into, but not beyond, the submucosa [22]. Patients were included if they were treated with surgical resection of T1 CRC. Transanal endoscopic microsurgery was considered an endoscopic treatment, as no lymphadenectomy is performed. Exclusion criteria were hereditary predisposition for CRC, inflammatory bowel disease, synchronous CRC (defined as CRC in the previous 5 years before detection of T1 CRC, or elsewhere in the colorectum at the time of detection of T1 CRC), non-CRC-related death within 1 year, non-adenocarcinoma, neo-adjuvant radiotherapy, unknown number of retrieved LNs, and AJCC stage IV T1 CRC at diagnosis.

This study was approved by the Medical Ethics Review Committee of the University Medical Center Utrecht (reference number 15-487/C) and was carried out in accordance with the Helsinki Declaration. The study conforms to the STROBE guideline for cohort studies [23].

### Endpoints

In each participating center, study variables were collected from the electronic medical records, and the corresponding endoscopy, surgery, pathology, and radiology reports. Primary endpoint was incidence of recurrent cancer, either local or distant. Local recurrence was defined as malignant tissue at the site of the anastomosis. Distant recurrence was defined as metastasis to extra-colonic organs, bone or peritoneum confirmed with imaging or histology. A new primary CRC elsewhere in the colon or rectum was defined as a metachronous lesion, not as recurrence. Secondary endpoint was prevalence of LNM at time of surgery, defined as positive LNs in the resection specimen as reported in the pathology report.

### Determinant and confounding factors

The determinant of interest was number of retrieved LNs, as reported in the pathology reports. We dichotomized LN yield using 10 retrieved LNs as cut-off, as this is the lowest recommended minimum for AJCC stage II CRC in current (inter)national guidelines [9, 11, 12].

Established risk factors (based on previous literature) for recurrent cancer and LNM were considered potential confounding factors [3, 4, 7, 24]. Data on potential clinical confounders were collected from the medical records and endoscopy reports, and included age, sex, tumor location (right colon vs. left colon vs. rectum), tumor size and tumor morphology (pedunculated vs. non-pedunculated) [3, 7, 24]. Right colon was defined as caecum, ascending and transverse colon including the splenic curve. Left colon was defined as the descending and sigmoid colon. Tumor morphology was defined as pedunculated if the presence of a stalk or Paris 0-Ip was reported in the endoscopy report [25]. Flat and sessile tumors were defined as non-pedunculated T1 CRCs [26]. Potential histological confounders were collected from the pathology reports, and included lymphovascular invasion (absent vs. present), depth of submucosal invasion (superficial vs. deep invasion), and grade of differentiation (good vs. moderate vs. poor) [4]. Deep submucosal invasion was defined as invasion depth ≥ 1 mm or sm2/3 for non-pedunculated T1 CRC, and Haggitt 4 for pedunculated T1 CRC [4]. For the endpoint recurrent cancer, the list of potential confounders was extended with LNM as a confounding factor [7].

Additional patient characteristics (not causally related to recurrent cancer or LNM and therefore not considered potential confounders) and follow-up characteristics were collected from medical records. Patient characteristics were body mass index (calculated as weight in kilograms divided by height in meters squared) and comorbidity according to the American Society of Anesthesiologists Physical Status classification [27]. Follow-up was performed according to routine clinical care. Follow-up started at the date of diagnosis and ended at the date of detection of recurrence, death, or last follow-up.

### Statistical analysis

Categorical data were expressed as frequencies and percentages; continuous variables as means with standard deviation (SD) or medians with interquartile range (IQR).

Primary aim of this study was to evaluate the association between number of retrieved LNs and recurrent cancer. Despite our large multicenter cohort of patients spanning an inclusion period of many years, the absolute number of recurrences was expected to be small, since recurrent cancer after surgical resection of T1 CRC is a rare event. We therefore used propensity scores allowing for the adjustment for more confounders than would have been feasible using standard multivariable adjustment approaches [28–30]. Inverse probability weighting (IPW) was used to account for baseline differences in predictive characteristics between patients with LN yield < 10 vs. ≥ 10. A propensity score was derived by fitting a logistic regression model with the dichotomized number of retrieved LNs as the dependent variable and potential confounders as predictors. We adjusted for confounders in a two-step approach. For the primary analysis, we adjusted for clinical confounding factors (i.e., age, sex, tumor location, tumor size, tumor morphology, and presence of LNM). Secondary supporting analyses were performed to additionally correct for histological confounding factors (i.e., lymphovascular invasion, depth of submucosal invasion, grade of differentiation), which were not included in the primary analysis as they were missing in a considerable number of cases (Table 1). Continuous variables (i.e., age and tumor size) were fitted in the propensity models by restricted cubic spline functions, dummies were used for categorical variables (no interaction terms). Then, the inverse of the (propensity score-derived) predicted probability for actual LN yield was used to weigh patients in a Cox regression model relating the dichotomized LN yield as the sole determinant of recurrent cancer, yielding hazard ratios (HR) for a high (≥10) vs. low (< 10) retrieval of LNs. Patients who did not develop recurrent cancer were censored at the last follow-up moment or date of death. No violation of the proportionality of the hazard assumption was observed following inspection of the scaled Schoenfeld residuals. Bootstrapping was performed to obtain two-sided *P* values and 95% confidence intervals (CI) for IPW-derived estimates.

**Table 1:** Baseline characteristics for patients with a LN yield < 10 vs. ≥ 10

	Baseline characteristics original cohort		Baseline characteristics LN yield < 10 vs. ≥ 10								
			Unadjusted data^a^			IPW adjusted data for the confounders considered for the primary analysis^a,c^			IPW adjusted data for the confounders considered for the secondary supporting analysis^a,d^		
	N = 1017^a^	Missing (%)^b^	LN yield < 10 N = 612	LN yield ≥ 10 N = 405	*P* value	LN yield < 10 N = 612	LN yield ≥ 10 N = 405	*P* value	LN yield < 10 N = 612	LN yield ≥ 10 N = 405	*P* value
Age in years	69.1	0.5	68.6	69.7	0.07	69.1	69.0	0.47	69.1	69.1	0.74
Male sex	54.6	0	55.7	52.8	0.40	54.6	54.0	0.30	54.5	53.7	0.35
BMI in kg/m^2^	26.9	27.9	26.9	27.0	0.70	26.8	27.0	0.54	26.7	27.1	0.46
ASA score		0.4									
- ASA I - ASA II - ASA III–IV	29.7 49.5 20.8		31.6 47.5 20.9	26.9 52.3 20.7	0.13 0.15 0.99	30.3 47.8 21.8	29.0 52.0 18.9	0.67 0.20 0.24	30.1 48.0 21.9	29.1 51.8 19.0	0.79 0.25 0.25
Tumor location		0									
- Right colon - Left colon - Rectum	24.1 58.8 17.1		15.2 68.6 16.2	37.5 44.0 18.5	<0.001 <0.001 0.38	24.2 58.7 17.0	24.1 58.8 17.1	0.62 0.89 0.67	23.9 59.0 17.1	23.9 58.9 17.2	0.84 0.83 0.85
Tumor morphology		5.0									
- Pedunculated - Non-pedunculated	33.4 66.6		39.3 60.7	24.1 75.9	<0.001 <0.001	33.3 66.7	33.7 66.3	0.39 0.39	33.4 66.6	33.7 66.3	0.68 0.68
Tumor size in cm	2.7	9.4	2.6	2.9	0.003	2.7	2.7	0.82	2.7	2.7	0.82
Lymph node metastasis	8.3	0	6.0	11.6	0.002	8.6	8.5	0.91	8.3	8.4	0.75
Adjuvant chemotherapy	4.4	0	3.6	5.7	0.15	4.6	4.5	0.92	4.4	4.6	0.82
Lymphovascular invasion		53.8									
- Absent - Present	79.2 20.8		70.1 29.9	79.8 20.2	0.04 0.04	69.7 30.3	78.9 21.1	0.03 0.03	73.3 26.7	73.2 26.8	0.70 0.70
Differentiation grade		18.5									
- Well - Moderate - Poor	14.0 80.6 5.4		14.9 79.4 5.7	12.7 82.3 5.0	0.44 0.31 0.63	15.1 79.6 5.4	12.9 82.0 5.1	0.47 0.41 0.59	14.1 80.6 5.3	14.2 80.7 5.1	0.71 0.73 0.62
Invasion depth		50.3									
- Haggitt I/II/III or SM1 - Haggitt IV or SM2/3	44.9 55.1		47.3 52.7	41.4 58.6	0.21 0.21	43.7 56.3	45.8 54.2	0.67 0.67	44.6 55.4	44.3 55.7	0.83 0.83

*ASA* American Society of Anesthesiologists, *BMI* body mass index, *﻿﻿cm* centimeter﻿﻿, *IPW* inverse probability weighting, *kg* kilogram, *LN* lymph node, *m* meter

^a^Values are means for continuous variables and percentages for categorical variables

^b^Percentage of patients with missing data in the original cohort

^c^Inverse probability weighting was based on age, sex, tumor location, tumor size, tumor morphology and the presence of lymph node metastasis (primary analysis)

^d^Inverse probability weighting was based on age, sex, tumor location, tumor size, tumor morphology, invasion depth, presence of lymphovascular invasion, differentiation grade, and presence of lymph node metastasis (secondary supporting analysis)

Assuming that a low LN yield risks understaging and these understaged patients would fail to receive chemotherapy that could prevent recurrence [31], we additionally repeated our main analysis restricted to patients in whom no LNM were observed [32]. Analyses were performed in the same manner with IPW analysis as described above, adjusting for the same potential confounders.

Furthermore, to assess the sensitivity of our results for the way we dichotomized LN yield in our analyses, the number of retrieved LNs was also analyzed continuously in univariable regression analyses. Moreover, sensitivity analyses were performed using 12 retrieved LNs as cut-off (using similar analysis as used for 10 retrieved LNs as cut-off), as this cut-off is often used as a quality measure for adequate staging in AJCC stage II CRC [9].

Several checks were performed to evaluate the appropriateness of the IPW analysis, namely (1) baseline characteristics before and after IPW adjustment for confounders were compared; (2) the *c*-index of the propensity models was assessed using the propensity model’s own IPWs (a *c*-index of 0.5 indicates a successfully obtained balance of confounders after adjusting; a *c*-index of 1.0 indicates extreme remaining imbalance); and (3) the maximum weight of a single patient used in the IPW-adjusted analysis to obtain balance in potential confounders was assessed as a quality instrument to assess whether a single or a few cases influence the risk estimate excessively [33, 34].

The secondary aim was to explore the association between LN yield and detection of LNM. Like recurrence, a low number of LNM were observed. Therefore, analyses were performed in the same manner with IPW analysis as described above with the presence of LNM as the endpoint, correcting for the same confounders except for the presence of LNM itself. Inverse probability weighted logistic regression analysis was used yielding odds ratios (OR) for a high (≥ 10) vs. low (< 10) retrieval of LNs on the outcome LNM.

Information on LN yield and the presence of LNM was available for all patients; however, several clinicopathological confounding variables had missing data (Table 1). As simply excluding patients with missing data is inefficient and increases the risk of selection bias, we used multiple imputation before data analysis [35]. Missing data was assumed to be missing at random. Multivariate imputation by chained equations (10 imputation datasets, 25 iterations, healthy convergence) was performed [36]. Rubin’s rules were used to pool results across imputation datasets [37]. Percentage of complete cases (i.e., percentage of patients with no imputed values) were reported for each analysis, together with the percentage of observed data points. For the primary outcome, a sensitivity analysis excluding patients with missing values was performed (complete-case analysis) to determine whether this agreed with the imputed results (Additional file 1: Table S1).

GraphPad Prism version 6.02 (GraphPad software Inc., San Diego, CA, USA) was used to draw figures. IBM SPSS Statistics version 21 (SPSS Inc., Chicago, IL, USA) and R version 3.2.2 (RStudio Inc., Boston, MA, USA) were used for statistical analysis. A two-sided *P* value < 0.05 was considered significant.

## Results

### Patient characteristics

**Fig. 1:**
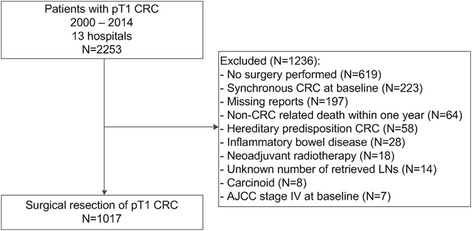
Study flowchart. *AJCC* American Joint Committee on Cancer; *CRC* colorectal cancer; *LN* lymph node

A total of 2253 patients with T1 CRC diagnosed between 2000 and 2014 were identified in the participating hospitals. A total of 1017 patients treated for pT1 CRC with surgical resection remained eligible for analysis (Fig. 1). Mean age of the cohort was 69.1 years (SD 9.6) and 54.6% of patients were male. A total of 405 patients (39.8%) had a LN yield ≥ 10. An overview of the baseline characteristics of patients with a LN yield < 10 vs. ≥ 10 before and after IPW adjustment for the confounders considered for the primary analysis (adjustment for clinical factors) and the secondary supporting analysis (adjustment for clinical and histological factors) is provided in Table 1. Before adjustment by IPW, patients with a LN yield ≥ 10 had T1 CRCs that were more often located in the right colon (37.5% vs. 15.2%, *P* < 0.001), had more often a non-pedunculated morphology (75.9% vs. 60.7%, *P* < 0.001), had a larger tumor size (2.9 vs. 2.6 cm, *P* = 0.003), more often had LNM (11.6% vs. 6.0%, *P* = 0.002), and less often showed lymphovascular invasion (20.2% vs. 29.9%, *P* = 0.04). Following adjustment, baseline characteristics between groups were comparable.

### LN yield in a routine clinical setting

**Fig. 2:**
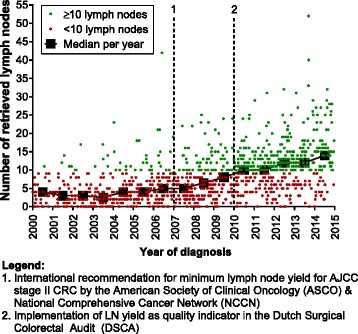
Scatterplot of LN retrieval in T1 CRC over the years. Each dot represents one patient. The squares indicate the median LN yield per year

Median LN yield was 7 (IQR 3–12; range 0–52). Median LN yield increased over time from 4 (IQR 2–8) from 2000 to 2009 (N = 574) to 11 (IQR 7–15) from 2010 to 2014 (N = 443) (*P* < 0.001). If a minimum of 10 LNs retrieved was considered an adequate resection, this threshold value was achieved in 19.5% (112/574) of patients treated before 2010 versus 66.1% (293/443) of patients treated from 2010 onwards (*P* < 0.001). A scatterplot of LN retrieval over the years is presented in Fig. 2.

### LN yield and recurrence

During the 4581 person-years of follow-up (median 49.0 months; IQR 19.6–81.5), 41 patients (4.0%) were diagnosed with recurrent cancer, corresponding to 9.0 events (95% CI 6.5–12.0) per 1000 person years of follow-up (Table 2). Recurrences occurred most frequently in distant organs (*N* = 23), followed by local (*N* = 11) or both local and distant recurrences (*N* = 7). Distant metastases were most often located in lung (*N* = 13) and liver (*N* = 16). Recurrences were detected after a median duration of 26.9 months (IQR 13.9–44.4).

**Table 2:** Recurrent cancer and LNM in T1 CRC, stratified for LN yield (< 10 vs. ≥ 10)

		Recurrent cancer			LNM
	N	N (%)	Total person-years of follow-up	Rate/1000 person-years of follow-up (95% CI)	N (%)
Total cohort	1017	41 (4.0)	4581	9.0 (6.5–12.0)	84 (8.3)
LN yield < 10	612	37 (6.0)	3403	10.9 (7.8–14.8)	37 (6.0)
LN yield ≥ 10	405	4 (1.0)	1179	3.4 (1.1–8.2)	47 (11.6)

*CI* confidence interval, *CRC* colorectal cancer, *LN* lymph node, *LNM* lymph node metastasis

Among 612 patients in whom < 10 LNs were retrieved, 37 patients (6.0%) developed recurrence. Among 405 patients in whom ≥ 10 LNs were retrieved, 4 patients (1.0%) developed recurrence (Table 2). In univariable analysis, LN yield ≥ 10 was associated with decreased risk for recurrence (unadjusted HR 0.27; 95% CI 0.10–0.76; *P* = 0.01) (Table 3). After adjustment for clinical confounders (i.e., age, sex, tumor location, tumor size, tumor morphology, and presence of LNM), LN yield ≥ 10 remained associated with a decreased risk for recurrence (adjusted HR 0.19; 95% CI 0.06–0.60; *P* = 0.005). Findings persisted after further adjusting for histological factors (adjusted HR 0.20; 95% CI 0.06–0.67; *P* = 0.009). Sensitivity analysis excluding patients with imputed values did not alter the outcomes (Additional file 1: Table S1).

**Table 3:** Unadjusted and adjusted association between LN yield (≥ 10 vs. < 10) and recurrent cancer after surgical resection of T1 CRC

		Hazard ratio (95% CI)	*P* value	Maximum (97.5^th^ percentile) IPW^c^	Post-IPW *c*-index^d^	Complete case (%)^e^	Observed data points (%)^f^
Total cohort N = 1017	Unadjusted	0.27 (0.10–0.76)	0.01	–	–	100	100
	Adjusted for clinical factors^a^	0.19 (0.06–0.60)	0.005	5.4 (4.4)	0.48	88	98
	Adjusted for clinical & histological factors^b^	0.20 (0.06–0.67)	0.009	7.1 (4.4)	0.50	18	88
LN negative patients N = 933	Unadjusted	0.25 (0.08–0.83)	0.02	–	–	100	100
	Adjusted for clinical factors^a^	0.21 (0.06–0.77)	0.02	5.6 (4.5)	0.48	88	98
	Adjusted for clinical & histological factors^b^	0.23 (0.06–0.81)	0.02	7.1 (4.5)	0.49	17	87

*CI* confidence interval, *CRC* colorectal cancer, *IPW* inverse probability weighting, *LN* lymph node

^a^Age (continuously), sex (male vs. female), tumor location (right colon vs. left colon vs. rectum), tumor size (continuously), tumor morphology (pedunculated vs. non-pedunculated) and lymph node metastasis (presence vs. absence) (the latter only in the total cohort, not in the analysis with LN-negative patients)

^b^Age (continuously), sex (male vs. female), tumor location (right colon vs. left colon vs. rectum), tumor size (continuously), tumor morphology (pedunculated vs. non-pedunculated), invasion depth (deep vs. superficial submucosal invasion), lymphovascular invasion (presence vs. absence), differentiation grade (poor vs. moderate vs. good), and lymph node metastasis (presence vs. absence) (the latter only in the total cohort, not in the analysis with LN-negative patients)

^c^The maximum weight of a single patient used in the IPW adjusted analysis to obtain balance in potential confounders. This is a quality instrument to assess whether a single or a few cases influence the risk estimate excessively. As a rule of thumb this should be lower than 10% of the analyzed dataset (i.e., smaller than 100 and 90 for the total cohort and LN-negative patients, respectively)

^d^This is an estimate of the balance of confounders after adjusting by inverse probability weighting (0.50 complete balance, 1.00 complete unbalance)

^e^Percentage of complete cases (i.e., cases with no imputed values for any of the evaluated variables of that analysis). Note: analysis was performed on the imputed dataset concerning all cases

^f^Percentage of available data points before imputation. Note: analysis was performed on the imputed dataset concerning all cases

**Fig. 3:**
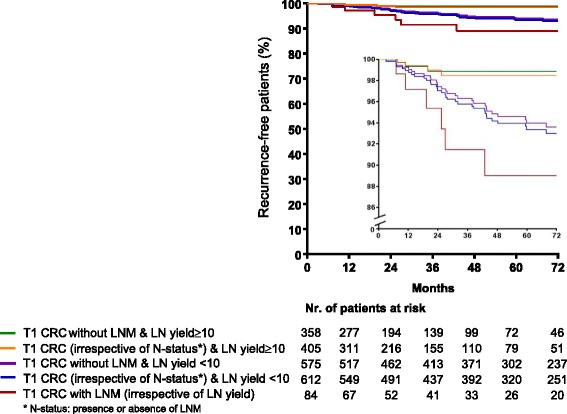
Kaplan–Meier curve of percentage of recurrence-free T1 CRC patients in relation to LN yield and presence of LNM. Green line: patients with T1 CRC without LNM and LN yield ≥ 10; orange line: patients with T1 CRC (with and without LNM) and LN yield ≥ 10; purple line: patients with T1 CRC without LNM and LN yield < 10; blue line: patients with T1 CRC (with and without LNM) and LN yield < 10; red line: patients with T1 CRC with LNM (irrespective of LN yield). The inset shows the same data on an enlarged y axis. Abbreviations: CRC: colorectal cancer; LN: lymph node; LNM: lymph node metastasis; nr: number

In patients without LNM (*N* = 933 patients; *N* = 35 recurrences), LN yield ≥ 10 remained associated with decreased risk for recurrence, also after adjusting for clinicopathological factors (adjusted HR 0.23; 95% CI 0.06–0.81; *P* = 0.02). Kaplan–Meier curves of recurrence-free patients stratified for LN yield (< 10 vs. ≥ 10) and LNM (presence vs. absence) are presented in Fig. 3.

To assess the sensitivity of our results for the way we dichotomized LN yield, sensitivity analyses were performed with the number of retrieved LNs without thresholds and using 12 LNs as cut-off. Without thresholds, LN yield remained associated with a decreased risk for recurrence (per retrieved LN: HR 0.90; 95% CI 0.84–0.97; *P* = 0.007). Of the 749 patients in whom < 12 LNs were retrieved, 37 patients (4.9%) developed recurrences. Of the 268 patients in whom ≥ 12 LNs were retrieved, 4 patients (1.5%) developed recurrences. Similar to a LN yield ≥ 10, the HR of a LN yield ≥ 12 pointed towards a decreased risk for recurrence, albeit non-significant (adjusted HR 0.41; 95% CI 0.12–1.34; *P* = 0.14) (Additional file 1: Table S2).

### LN yield and LNM

LNM were detected in 84 patients, corresponding to a prevalence of 8.3% (84/1017) (Table 2); 1 positive LN was detected in 54 patients, 2 in 18 patients, 3 in 4 patients, and ≥ 4 in 8 patients, with a maximum of 8 positive LNs per patient. In 6 patients with LNM, recurrence occurred during follow-up. In univariable analysis, the number of retrieved LNs (without thresholds) was associated with increased detection of LNM (per retrieved LN: OR 1.04; 95% CI 1.01–1.07; *P* = 0.006).

LNM were detected in 37 patients (6.0%) with a LN yield < 10 and in 47 patients (11.6%) with a LN yield ≥ 10 (Table 2). In univariable analysis, LN yield ≥ 10 was positively associated with increased detection of LNM (unadjusted OR 2.04; 95% CI 1.30–3.20; *P* = 0.002) (Table 4). After adjusting for clinical confounding factors, LN yield ≥ 10 remained associated with increased detection of LNM (adjusted OR 2.09; 95% CI 1.28–3.42). Further adjusting for histological confounding factors did not alter the outcomes (adjusted OR 2.27; 95% CI 1.39–3.69; *P* = 0.001).

**Table 4:** Unadjusted and adjusted association between LN yield (≥ 10 vs. < 10) and detection of LNM in T1 CRC

	Odds ratio (95% CI)	*P* value	Maximum (97.5^th^ percentile) IPW^c^	Post-IPW *c*-index^d^	Complete case (%)^e^	Observed data points (%)^f^
Unadjusted	2.04 (1.30–3.20)	0.002	–	–	100	100
Adjusted for clinical factors^a^	2.09 (1.28–3.42)	0.003	5.1 (4.2)	0.48	88	98
Adjusted for clinical and histological factors^b^	2.27 (1.39–3.69)	0.001	6.6 (4.3)	0.49	18	85

*CI* confidence interval, *﻿CRC*: colorectal cancer﻿,*IPW* inverse probability weighting, *LN* lymph node, ﻿*LNM* lymph node metastasis﻿

^a^Age (continuously), sex (male vs. female), tumor location (right colon vs. left colon vs. rectum), tumor size (continuously), and tumor morphology (pedunculated vs. non-pedunculated)

^b^Age (continuously), sex (male vs. female), tumor location (right colon vs. left colon vs. rectum), tumor size (continuously), tumor morphology (pedunculated vs. non-pedunculated), invasion depth (deep vs. superficial submucosal invasion), lymphovascular invasion (presence vs. absence), and differentiation grade (poor vs. moderate vs. good)

^c^The maximum weight of a single patient used in the IPW adjusted analysis to obtain balance in potential confounders. This is a quality instrument to assess whether a single or a few cases influence the risk estimate excessively. As a rule of thumb this should be lower than 10% of the analyzed dataset (i.e., smaller than 100)

^d^This is an estimate of the balance of confounders after adjusting by inverse probability weighting (0.50 complete balance, 1.00 complete unbalance)

^e^Percentage of complete cases (i.e., cases with no imputed values for any of the evaluated variables of that analysis). Note: analysis was performed on the imputed dataset concerning all cases

^f^Percentage of available data points before imputation. Note: analysis was performed on the imputed dataset concerning all cases

## Discussion

To the best of our knowledge, this is the first large-scale study evaluating the association between LN yield and long-term recurrence rates in patients with T1 CRC when adjusting for multiple confounding factors. A LN yield < 10 was associated with an increased risk for recurrence after surgical resection of T1 CRC, even when adjusting for clinical and histological characteristics. Furthermore, LN yield ≥ 10 was independently associated with an increased detection of LNM. These findings underline the importance of performing an appropriate oncologic resection of the draining LNs and diligent LN search by the pathologist when patients with T1 CRC at high-risk for LNM are referred for surgical resection and question the legitimacy of a limited resection for these patients.

In contrast to earlier smaller studies, we validated a cut-off (≥ 10) that was chosen based on current recommendations for AJCC stage II CRC, instead of evaluating multiple study-specific cut-off points and selecting the most suitable one [16, 20]. This approach is less prone to the introduction of overestimation of the difference in outcomes between groups, and enhances the generalizability and external validity of the results [38]. To assess the sensitivity of our results for the manner in which the LN yield was dichotomized, we performed sensitivity analyses with the number of retrieved LNs analyzed continuously and additionally used 12 retrieved LNs as the cut-off. In all analyses, the HR indicated a decreased risk for recurrence.

Our findings build on a study conducted with data from the Surveillance, Epidemiology, and End Results cancer registry in patients with AJCC stage I CRC, comprising both T1N0 and T2N0 CRC [31]. An increased LN yield was associated with improved overall survival. However, this study used a population-based registry, and could therefore only evaluate the association between LN yield and mortality due to all causes. As the vast majority (≥ 95%) of patients with T1 CRC without LNM do not die as a result of CRC, recurrence rates rather than survival rates are informative when evaluating factors associated with prognosis in this early stage [5, 6].

Several explanations can be hypothesized for the observed association with recurrence. The first is understaging, with positive nodes missed when an insufficient number of LNs is retrieved. Consequently, chemotherapy will not be considered in these patients and the missed residual cancer cells may metastasize to distant organs. Evidence on the benefit of chemotherapy in patients with T1 CRC with LNM is limited. However, it is currently advised in these patients [39]. Our finding that ≥ 10 retrieved LNs resulted in a significantly higher percentage of nodal positive patients supports this explanation, as was also demonstrated in previous work for advanced CRC stages [31, 40]. However, a population-based retrospective observational study questioned this mechanism as the main cause of the observed association, as patients with higher LN yields were only slightly more likely to have LNM, suggesting that some other unmeasured factors resulted in better patient outcomes [8]. It has been hypothesized that the number of retrieved LNs might reflect tumor biology [41]. The tumor microenvironment and the host’s immune response have been shown to be of major importance in tumor progression [42]. Thus, a higher LN yield may reflect a stronger immune response, reducing the risk for recurrence.

Some limitations should be mentioned. Although the present cohort is one of the largest on T1 CRCs to date with long-term follow-up enabling the evaluation of the association between LN yield and recurrence when adjusting for multiple confounding factors, it is an observational study on a retrospective cohort. Inherent to the design, we had to deal with missing data. Information on histological potential confounders was missing in a considerable number of patients, resulting in one or more variables having to be imputed in more than 82% of patients in the secondary supporting analysis. Nevertheless, the estimates resulting from the secondary analyses with and without imputation were similar, suggesting that selection bias due to missingness – if present – was rather limited (results without imputation can be found in Additional file 1: Table S1). Furthermore, due to the retrospective design, no data was available on the effort of the surgeon for adequate lymphadenectomy, such as the extent of the mesentery excised, and no standardized pathologic evaluation of the resection specimen was performed. This could have shed some light on the role of the surgeon and pathologist on the LN yield. However, the primary aim of this study was to explore the association between LN yield and recurrence in T1 CRC, not to explore the influence of the pathologist and surgeon, as has been done in previous studies [17, 43–46]. Taking the results of these studies into account, we believe LN yield is a shared responsibility of both surgeon and pathologist.

Secondly, although we corrected as efficiently as possible for the measured confounders, it remains unclear how well the unmeasured confounders have been adjusted for. New predictive markers in CRC have been identified in the past two decades, including tumor budding as prognostic marker for LNM and macroscopic pathological assessment of the quality of the circumferential resection margin (CRM) as a prognostic marker for recurrence in rectal cancer [4, 47, 48]. Standardized pathologic reporting of the quality of the CRM was only introduced in Dutch guidelines in recent years, and consensus on the standardized assessment of tumor budding has only recently been achieved (International Tumor Budding Consensus Conference 2016) [49]. Therefore, this hampered the evaluation of these factors in our analysis. To explore the magnitude of potential bias introduced by lack of information on the quality of the CRM, we repeated the main analysis when excluding patients with rectal T1 cancer, yielding similar results, suggesting that the introduced potential bias is limited (Additional file 1: Table S3).

Thirdly, we were not informed on tumor biology such as genetic mutations, microsatellite instable status, or consensus molecular subtype classification [50, 51]. However, similar to data from the Surveillance, Epidemiology, and End Results cancer registry, we observed an increased LN yield over the years, suggesting that the observed survival benefit cannot be completely attributed to tumor biology [8, 9]. It is not unlikely that the Dutch Surgical Colorectal Audit, introduced in 2008, and the implementation of LN yield as a quality indicator in 2009 have contributed to the observed increment in LN yield from 2010 onwards. This finding shows that retrieving higher number of LNs is achievable in daily clinical practice, and should be aimed for. Moreover, a previous study observed that LN retrieval differed between different types of hospitals, suggesting that other quality issues may influence LN retrieval [52].

Finally, it should be emphasized that, despite our multicenter cohort spanning an inclusion period of many years, the absolute number of patients with recurrent cancer or LNM was still low. Although we resorted to statistical analysis techniques suited for such data and performed several checks to evaluate the appropriateness of our analysis, the low number of events unavoidably resulted in relatively broad CIs.

## Conclusion

In conclusion, in this observation cohort study, the retrieval of less than 10 LNs was associated with an increased risk for recurrence and decreased detection of LNM in T1 CRC. Given that gastroenterologists, surgeons and pathologists will all encounter T1 CRCs with increasing frequency due to the introduction of CRC screening programs, awareness on the consequences of an inadequate LN retrieval is of utmost importance.

## Additional file

Additional file 1: Table S1. Sensitivity analysis comparing the complete case hazard ratio and the multiple imputation hazard ratio for the association between lymph node (LN) yield ≥ 10 versus < 10 and the primary outcome recurrent cancer. **Table S2**. Unadjusted and adjusted association between LN yield ≥ 12 (*N* = 268 patients, *N* = 4 recurrences) versus LN yield < 12 (*N* = 749 patients, *N* = 37 recurrences) and recurrent cancer after surgical resection of T1 colorectal cancer. **Table S3**. Main analysis restricted to patients with T1 colon cancer (843 T1 colon cancers with 29 recurrences) in order to explore the magnitude of potential bias introduced by lack of information on the quality of the circumferential resection margin in rectal T1 cancer. (DOCX 18 kb)

## Abbreviations

AJCC: American Joint Committee on Cancer
CI: confidence interval
CRC: colorectal cancer
CRM: circumferential resection margin
HR: hazard ratio
IPW: inverse probability weighting
IQR: interquartile range
LN: lymph node
LNM: lymph node metastasis
OR: odds ratio
SD: standard deviation
