# Supplementary material for: The prognostic value of lymph node yield in the earliest stage of colorectal cancer: a multicenter cohort study
Source: BMC Med. 2017 Jul 14;15:129. doi: 10.1186/s12916-017-0892-7 (PMC5512847; doi:10.1186/s12916-017-0892-7)
Supplement: Additional file 1: Table S1. — Sensitivity analysis comparing the complete case hazard ratio and the multiple imputation hazard ratio for the association between lymph node (LN) yield ≥ 10 versus < 10 and the primary outcome recurrent cancer. Table S2. Unadjusted and adjusted association between LN yield ≥ 12 (N = 268 patients, N = 4 recurrences) versus LN yield < 12 (N = 749 patients, N = 37 recurrences) and recurrent cancer after surgical resection of T1 colorectal cancer. Table S3. Main analysis restricted to patients with T1 colon cancer (843 T1 colon cancers with 29 recurrences) in order to explore the magnitude of potential bias introduced by lack of information on the quality of the circumferential resection margin in rectal T1 cancer. (DOCX 18 kb) [file 12916_2017_892_MOESM1_ESM.docx]

**Additional file 1**

**Table S1.** Sensitivity analysis comparing the complete case hazard ratio (HR) and the multiple imputation HR for the association between LN yield ≥10 vs. <10 and the primary outcome recurrent cancer

|  | **% of complete cases** | **Multiple imputation HR** | **Complete case HR** |
| --- | --- | --- | --- |
| Unadjusted | 100% | HR 0.27 | HR 0.27 |
| Adjusted for clinical factors ^a^ | 88% | HR 0.19 | HR 0.23 |
| Adjusted for clinical & histological factors ^b^ | 18% | HR 0.20 | HR 0.23 |
| Abbreviations: HR: hazard ratio   1. Similar clinical confounder as main analysis (see legend Table 3 of article) 2. Similar clinical and histological confounders as main analysis (see legend Table 3 of article) | | | |

**Table S2.** Unadjusted and adjusted association between LN yield ≥12 (N=268 patients, N=4 recurrences) vs. LN yield<12 (N=749 patients, N=37 recurrences) and recurrent cancer after surgical resection of T1 CRC

|  | **HR (95%CI)** | **P-value** | **Complete case (%)** ^c^ |
| --- | --- | --- | --- |
| Unadjusted | 0.51 (0.18 – 1.44) | 0.20 | 100 |
| Adjusted for clinical factors ^a^ | 0.40 (0.12 – 1.26) | 0.12 | 88 |
| Adjusted for clinical & histological factors ^b^ | 0.41 (0.12 – 1.34) | 0.14 | 18 |
| Abbreviations: CI: confidence interval; HR: hazard ratio   1. Similar clinical confounder as main analysis (see legend Table 3 of article) 2. Similar clinical and histological confounders as main analysis (see legend Table 3 of article) 3. Percentage of complete cases which could be analyzed | | | |

**Table S3.** Main analysis restricted to patients with T1 colon cancer (843 T1 colon cancers with 29 recurrences), in order to explore the magnitude of potential bias introduced by lack of information on the quality of the circumferential resection margin in rectal T1 cancer.

| **Association between LN yield (≥10 vs. <10) and recurrent cancer after surgical resection of T1 colon cancer** | | | |
| --- | --- | --- | --- |
|  | **HR (95%CI)** | **P-value** | **Complete case (%)**^c^ |
| Unadjusted | 0.30 (0.09 – 1.00) | 0.05 | 100 |
| Adjusted for clinical factors ^a^ | 0.22 (0.06 – 0.80) | 0.02 | 88 |
| Adjusted for clinical & histological factors ^b^ | 0.24 (0.07 – 0.89) | 0.03 | 17 |
| Abbreviation: HR: hazard ratio; LN: lymph node   1. Similar clinical confounder as main analysis (see legend Table 3 of article) 2. Similar clinical and histological confounders as main analysis (see legend Table 3 of article) 3. Percentage of complete cases which could be analyzed | | | |
